# Gender-Affirming Hormone Treatment and Metabolic Syndrome Among Transgender Veterans

**DOI:** 10.1001/jamanetworkopen.2024.19696

**Published:** 2024-07-02

**Authors:** Leila Hashemi, Andriana Marijic Buljubasic, Matthew J. Budoff, Laurel A. Copeland, Nicholas J. Jackson, Guneet K. Jasuja, Jeffery Gornbein, Karen Reue

**Affiliations:** 1 VA Greater Los Angeles Health Care System, Department of General Internal Medicine, David Geffen School of Medicine, Los Angeles, California; 2 Yale School of Public Health, New Haven, Connecticut; 3 Department of Medicine, Lundquist Institute at Harbor-UCLA, Torrance, California; 4 VA Central Western Massachusetts Health Care System, Leeds; 5 Statistics Core, Department of Medicine, David Geffen School of Medicine, University of California, Los Angeles; 6 Center for Healthcare Organization & Implementation Research, US Department of Veterans Affairs, VA Bedford Health Care System, Bedford, Massachusetts; 7 Section of General Internal Medicine, Chobanian and Avedisian School of Medicine, Boston University, Boston, Massachusetts; 8 Department of Health Law, Policy and Management, School of Public Health, Boston University, Boston, Massachusetts; 9 Human Genetics, David Geffen School of Medicine, University of California, Los Angeles

## Abstract

**Importance:**

Gender-affirming hormone treatment (GAHT) is a common therapy for transgender individuals to reduce gender dysphoria and improve quality of life. Clarifying the long-term effects of GAHT remains a priority in transgender health research.

**Objective:**

To explore whether sex hormones (estradiol and testosterone) are associated with the development of metabolic syndrome in transgender veterans compared with cisgender veterans.

**Design, Setting, and Participants:**

This retrospective, longitudinal cohort study used *International Classification of Diseases, Ninth Revision* and *International Statistical Classification of Diseases and Related Health Problems, Tenth Revision* diagnosis codes for gender dysphoria from the Veterans Health Administration national database to identify transfeminine and transmasculine veterans receiving documented feminizing (estradiol) or masculinizing (testosterone) treatment from January 1, 2006, to December 31, 2019, and for whom the GAHT initiation date and metabolic syndrome component–related data were available. Transgender veterans were matched to cisgender referents.

**Exposure:**

Gender-affirming hormone treatment.

**Main Outcomes and Measures:**

Metabolic syndrome z-scores were calculated based on body mass index, systolic blood pressure, and levels of high-density lipoprotein cholesterol, triglycerides, and blood glucose. Changes in mean z-scores were compared among the transgender and cisgender groups before and after the index date (corresponding to GAHT initiation) using a repeated-measures analysis of variance model.

**Results:**

The cohort included 1290 participants: 645 transgender (494 [38.3%] transfeminine, 151 [11.7%] transmasculine) and 645 cisgender (280 [21.7%] female, 365 [28.3%] male). Mean (SD) age at the index date was 41.3 (13.2) years. Metabolic syndrome z-scores changed significantly over time and differed significantly across groups. Overall, transmasculine veterans had the greatest percentage increase in mean (SEM) z-scores after vs before the index date (298.0% [57.0%]; *P* < .001), followed by cisgender females (108.3% [27.5%]; *P* < .001), cisgender males (49.3% [27.5%]; *P* = .02), and transfeminine persons (3.0% [10.7%]; *P* = .77).

**Conclusions and Relevance:**

In this cohort study, in both cisgender and transgender veterans, estradiol was associated with reduced metabolic syndrome risk, whereas testosterone was associated with increased risk. However, transmasculine individuals had the greatest risk and transfeminine individuals had the lowest risk of metabolic syndrome associated with these hormones. This is relevant for the management of metabolic syndrome risk factors in cisgender and transgender individuals and to potentially predict the risk of atherosclerotic cardiovascular disease, type 2 diabetes, systolic hypertension, insulin resistance, and nonalcoholic fatty liver disease.

## Key Points

**Question:**

Is gender-affirming hormone treatment (GAHT) by proxy steroid associated with development and progression of metabolic syndrome?

**Findings:**

In this cohort study of 645 transgender veterans matched with 645 cisgender veterans, transmasculine veterans receiving testosterone had the greatest increase in metabolic syndrome z-scores after vs before the date of GAHT initiation, followed by cisgender females and cisgender males, while transfeminine participants receiving estradiol had the smallest change.

**Meaning:**

The findings suggest that testosterone is associated with a greater risk of development and progression of metabolic syndrome, particularly in transgender individuals.

## Teaser

This cohort study investigates associations between gender-affirming hormone treatment and development and progression of metabolic syndrome among transgender veterans compared with cisgender veterans.

## Introduction

An estimated 0.4% to 0.6% of adults in the US identify as transgender, meaning their gender identity differs from the sex assigned at birth, and this number is increasing.^1,2^ Many transgender individuals receive gender-affirming hormone treatment (GAHT) to reduce gender dysphoria and improve quality of life.^3^ Estrogen and antiandrogen hormone replacement is the main form of GAHT for transfeminine individuals, while testosterone replacement is the primary hormone treatment for transmasculine individuals.^4^

Metabolic syndrome refers to a collection of intertwined factors that are directly associated with increased risk of adverse outcomes, including atherosclerotic cardiovascular disease (ASCVD), insulin resistance, type 2 diabetes (T2D), systolic hypertension, and nonalcoholic fatty liver disease (NAFLD).^5,6^ Metabolic syndrome is typically diagnosed based on abnormal values relative to appropriate cutoff values for at least 3 of the 5 clinical measures: blood pressure (BP), high-density lipoprotein (HDL) cholesterol, triglycerides, blood glucose, and waist circumference.

The binary approach to metabolic syndrome classification makes it difficult to assess worsening of this condition over time. Gurka et al^7,8,9^ developed a novel sex- and race-specific metabolic syndrome risk continuous score, which is superior to a binary indicator for metabolic syndrome prediction.

Sex hormones play an important role in regulating fat distribution, weight, and triglycerides and fatty acid homeostasis such that they can influence metabolic syndrome development or regression.^10,11,12,13,14,15,16^ Conditions that cause females to experience reduced estradiol levels, such as menopause or ovariectomy, or increased testosterone levels, such as polycystic ovary syndrome, may promote metabolic syndrome development.^12,14,17^ In males, testosterone is converted to dihydrotestosterone by 5α-reductase and dihydrotestosterone is converted to estradiol by aromatase. Males with decreased aromatase levels experience greater risk of abdominal obesity, elevated blood lipid levels, and insulin resistance.^13,18,19^ These metabolic changes are also observed in males receiving antiandrogen treatment for prostate cancer and in males with Klinefelter syndrome (XXY sex chromosomes).^20,21^ XX chromosome–associated increased risk of metabolic syndrome is further supported by mouse model studies showing that presence of 2 X chromosomes in male mice promotes increased body fat and elevated plasma cholesterol levels.^22,23^

Multiple studies have investigated the association of GAHT with the individual components of metabolic syndrome and implicated GAHT in metabolic syndrome,^6,24^ but data regarding the longitudinal effects of GAHT on metabolic syndrome development and progression are lacking. The current study investigated the longitudinal association of GAHT with changes in metabolic syndrome z-scores in transfeminine and transmasculine individuals compared with cisgender males and females not receiving exogenous sex hormones. The analyses also explored whether the action of exogenous hormones was associated with chromosomes and/or organizational effects of sex hormones that occurred during development.

## Methods

This was a retrospective, longitudinal cohort study of transgender and cisgender veterans from the Veterans Health Administration (VHA). The institutional review board (IRB) of the VA Greater Los Angeles Health Care System reviewed and approved the study and waived informed consent because we used only retrospective data and did not have any contact with the participants. We followed the Strengthening the Reporting of Observational Studies in Epidemiology (STROBE) reporting guideline to report our research data.

### Cohort Ascertainment and Data Collection

The source population consisted of patients from US VHA databases with any *International Classification of Diseases, Ninth Revision (ICD-9)* or *International Statistical Classification of Diseases and Related Health Problems, Tenth Revision (ICD-10)* codes corresponding to gender identity disorder, gender dysphoria, or transsexualism (*ICD-9*: 302.3, 302.5, 302.6, or 302.8; *ICD-10*: F64, F64.0, F64.1, F64.8, F64.9, F65.1, or Z87.8) from January 1, 2006, to December 31, 2019 (eMethods in Supplement 1).^25^ To confirm gender identity status of study participants and obtain the feminizing (estradiol) or masculinizing (testosterone) hormone initiation date, we obtained the IRB’s permission to access and review patients’ medical records.

Demographics and comorbidities were extracted from VHA and Centers for Medicare & Medicaid Services (CMS) databases.^26^ The race and ethnicity data provided for this cohort were self-reported and were included in the analysis because the z-score equation is race dependent. Categories were Hispanic, non-Hispanic Black (hereafter, *Black*), non-Hispanic White (hereafter, *White*), and other (included Asian, Pacific Islander, unknown, and those who declined to answer). A combination of 2 outpatient or 1 inpatient *ICD-9* or *ICD-10* diagnostic or *Current Procedural Terminology* code was used to determine preexisting comorbidity status using the VHA and CMS datasets to improve accuracy.^26,27^ Laboratory results; prescription medications, including sex hormones; body mass index (BMI; calculated as weight in kilograms divided by height in meters squared); and BP were obtained from the VHA Corporate Data Warehouse; waist circumference data were not available. Using the date of hormonal transition as the index date, we accessed data up to 13 years before and 15 years after hormonal transition. Transgender and cisgender participants were matched for race and ethnicity, birth date, and index visit date using 1:1 matching cisgender individual for each transgender participant. Cisgender matches were selected from a pool of 17 000 veterans who had complete BMI, HDL, systolic BP (SBP), triglyceride, and blood glucose data obtained at a proxy index date (we used an outpatient visit within the same quarter of the transgender match’s index date). For a given transgender participant, the corresponding cisgender participant had the same race and ethnicity and was selected as the individual with the closest birth date to that of the transgender participant and with laboratory data obtained closest to the hormone transition in the corresponding transgender participant (ie, index date). All birth dates and index dates matched to within 1 year or better. Cisgender participants were not receiving any external sex hormones.

### Metabolic Syndrome z-Score Calculations

The metabolic syndrome z-score was adopted from BMI-based equations by Gurka et al.^9^ These equations are sex- and race-dependent (White and Black race). We used sex assigned at birth to calculate the metabolic syndrome z-scores before and after transition. We used the formula for White individuals for any participants in the “other” race and ethnicity category, as there were no corresponding formulas for these categories. Time in years was computed from the index date for each transgender participant and the corresponding date for the matched cisgender individual. Year 0 was defined as the year of hormonal transition, corresponding to the year initiated by the index date. Years (and observations) before the hormone transition thus have negative values, such that year −3 corresponds to 3 years before year 0, the transition year, and year 4 is the fourth year after year 0. The standardized metabolic syndrome scores and their SEMs were compared among the 4 groups (transfeminine, transmasculine, cisgender female, and cisgender male) over time (years) before and after transition. The mean values of metabolic syndrome components before and after the index date were calculated using the same model.

### Statistical Analysis

Patient characteristics were reported using means and SDs for continuous variables and frequencies and percentages for categorical variables. The mean profiles were computed using a repeated measure (mixed) analysis of variance model. A mixed model was particularly necessary since no participant had the full surveillance time (13 years before to 15 years after the index date) (eTable in Supplement 1). This model included random participant effects to allow for autocorrelation. When participants had several observations across time, the model could compute a correlation-covariance matrix and use this matrix and the data to reconstruct a maximum likelihood estimate of the true mean profile that was unbiased if the incompleteness was not systematic (ie, not much more likely to be missing before vs after transition). The resulting mean profile was an estimate of what would have been observed if each participant had complete follow-up. These models also allowed for the assessment of correlations among multiple observations across time for the same participants. A check of the residual errors confirmed that the errors followed a normal distribution. Plots of smoothed mean profiles were computed using the locally estimated scatterplot smoothing method. These linear mixed models allowed for the modeling of heterogeneity in the change in metabolic syndrome z-scores across time.

All analyses were conducted using SAS, version 8.3 (SAS Institute Inc). Two-sided *P* < .05 was considered significant.

## Results

### Characteristics of Study Participants

Among 11 011 veterans, 999 medical records were reviewed (eFigure in Supplement 1); 789 individuals had a gender dysphoria diagnosis. The analytic dataset for this study included 1290 participants, of whom 645 were receiving GAHT (494 [38.3%] transfeminine and 151 [11.7%] transmasculine) and 645 were not receiving exogenous sex hormone treatment (280 [21.7%] cisgender female and 365 [28.3%] cisgender male) (Table 1). A total of 106 participants (8.2%) were Black; 108 (8.4%), Hispanic; 942 (73.0%), White; and 134 (10.4%), other race and ethnicity. Mean (SD) age of the cohort at the index date was 41.3 (13.2) years. The median surveillance time was 6 years (IQR, 3-11 years), and maximum was 15 years (eTable in Supplement 1). Table 1 shows the time-adjusted mean values at the index date for metabolic syndrome components (including BMI, SBP, HDL, log triglycerides, and blood glucose) for each of the 4 groups.

**Table 1.** Participant Baseline Characteristics

Characteristic	Participants (N = 1290)^a^			
	Transfeminine (n = 494)	Transmasculine (n = 151)	Cisgender female (n = 280)	Cisgender male (n = 365)
Race and ethnicity				
Hispanic	48 (9.7)	6 (4.0)	20 (7.1)	34 (9.3)
Non-Hispanic Black	23 (4.7)	30 (19.9)	27 (9.6)	26 (7.1)
Non-Hispanic White	377 (76.3)	94 (62.3)	203 (72.5)	268 (73.4)
Other^b^	46 (9.3)	21 (13.9)	30 (10.7)	37 (10.1)
Age at index date, mean (SD), y	41.3 (13.2)	41.3 (13.2)	41.4 (13.4)	41.4 (13.4)
Metabolic syndrome components at index date, mean (SD)				
BMI	29.70 (6.10)	29.20 (5.71)	28.94 (0.37)	30.6 (6.13)
SBP, mm Hg	127.60 (13.20)	125.70 (14.60)	126.10 (15.80)	127.0 (13.90)
HDL level, mg/dL	44.20 (13.20)	48.70 (16.90)	47.40 (12.30)	45.20 (13.10)
Log triglycerides, mg/dL	178.30 (169.80)	143.70 (126.60)	154.40 (102.60)	163.30 (119.30)
Blood glucose level, mg/dL	112.80 (45.60)	98.50 (36.30)	102.40 (33.70)	106.50 (34.50)

Abbreviations: BMI, body mass index (calculated as weight in kilograms divided by height in meters squared); HDL, high-density lipoprotein; SBP, systolic blood pressure.

SI conversion factors: To convert blood glucose to mmol/L, multiply by 0.0555; HDL to mmol/L, multiply by 0.0259; triglycerides to mmol/L, multiply by 0.0113.

^a^ Data are presented as number (percentage) of participants unless otherwise indicated.

^b^ Included Asian, Pacific Islander, unknown, and those who declined to answer.

### Longitudinal Changes in Metabolic Syndrome z-Score

Table 2 presents the mean changes in unadjusted metabolic syndrome components before vs after index dates (13 years prior to the index date and up to 15 years after the index date) and comparisons among groups. Transmasculine participants had the greatest mean (SEM) increase in BMI after transition (2.3 [0.6]; *P* < .001), whereas BMI did not change significantly in transfeminine individuals (0.3 [0.2]; *P* = .13). Transmasculine participants had a significant reduction in HDL levels (mean [SEM], −3.8 [1.8] mg/dL; *P* = .03) (to convert to mmol/L, multiply by 0.0259), while transfeminine individuals had the greatest increase in HDL concentration (mean [SEM], 7.3 [0.7] mg/dL; *P* < .001). Individuals in the transgender groups showed significant percentage changes in log triglycerides values, which were decreased in transfeminine participants (mean [SEM], −0.09 [0.03] mg/dL; *P* = .005) and increased in transmasculine participants (mean [SEM], 0.28 [0.08] mg/dL; *P* < .001). All groups had significant increases in blood glucose values from before to after the index date, with the greatest increase in the transmasculine group (mean [SEM], 26.7 [7.0] mg/dL; *P* < .001) (to convert to mmol/L, multiply by 0.0555). The mean (SEM) SBP values (shown in Table 2) increased in transmasculine (6.0 [2.1] mm Hg; *P* = .004) and cisgender male (4.4 [0.9] mm Hg; *P* < .001) individuals, while it did not change significantly in transfeminine and cisgender female individuals. Overall, transmasculine veterans had the greatest percentage increase in mean (SEM) z-scores after vs before the index date (298.0% [57.0%]; *P* < .001), followed by cisgender females (108.3% [27.5%]; *P* < .001), cisgender males (49.3% [27.5%]; *P* = .02), and transfeminine persons (3.0% [10.7%]; *P* = .77).

**Table 2.** Within-Group Changes Before and After GAHT and Between-Group Differences in Within-Group Change

Metabolic syndrome component	Change, mean (SEM)				Difference-in-difference *P* value^a^					
	CF	CM	TF	TM	CF vs CM	CF vs TF	CF vs TM	CM vs TF	CM vs TM	TF vs TM
**BMI**										
Before GAHT	28.2 (0.4)	29.1 (0.3)	29.6 (0.4)	27.7 (0.8)	.29	.001	.24	.01	.08	.004
After GAHT	29.6 (0.4)	30.1 (0.3)	30.0 (0.3)	30.0 (0.6)						
Difference	1.4 (0.3)	1.1 (0.2)	0.3 (0.2)	2.3 (0.6)						
*P* value^b^	<.001	<.001	.13	<.001						
**HDL level, mg/dL**										
Before GAHT	45.8 (1.0)	44.6 (0.8)	42.6 (0.9)	53.1 (1.9)	.18	<.001	<.001	.002	<.001	<.001
After GAHT	48.7 (0.8)	49.1 (0.7)	49.9 (0.7)	49.4 (1.4)						
Difference	2.9 (0.9)	4.4 (0.7)	7.3 (0.7)	−3.8 (1.8)						
*P* value^b^	.001	<.001	<.001	.03						
**Log triglycerides, mg/dL** ^c^										
Before GAHT	4.79 (0.04)	4.86 (0.03)	4.97 (0.04)	4.44 (0.08)	.08	.003	.02	.15	<.001	<.001
After GAHT	4.86 (0.03)	4.83 (0.03)	4.88 (0.03)	4.72 (0.06)						
Difference	0.07 (0.04)	−0.02 (0.03)	−0.09 (0.03)	0.28 (0.08)						
*P* value^b^	.10	.47	.005	<.001						
**Blood glucose level, mg/dL**										
Before GAHT	97.2 (3.4)	97.3 (2.4)	103.8 (2.8)	92.7 (6.1)	.07	.71	.06	.10	.41	.08
After GAHT	108.2 (2.4)	116.2 (2.2)	116.5 (2.1)	117.5 (4.1)						
Difference	11.0 (3.5)	19.0 (2.6)	12.7 (2.7)	24.8 (6.5)						
*P* value^b^	.001	<.001	<.001	<.001						
**SBP, mm Hg**										
Before GAHT	124.6 (1.1)	124.0 (0.8)	127.2 (0.9)	117.5 (2.0)	.04	.08	.06	<.001	.49	.002
After GAHT	126.1 (0.8)	128.4 (0.7)	126.2 (0.7)	123.5 (1.3)						
Difference	1.5 (1.1)	4.4 (0.9)	−1.0 (0.9)	6.0 (2.1)						
*P* value^b^	.18	<.001	.27	.004						
**Metabolic syndrome z-score**										
Before GAHT	0.29 (0.08)	0.28 (0.06)	0.56 (0.07)	−0.26 (0.15)	.08	.003	.005	.16	<.001	<.001
After GAHT	0.60 (0.07)	0.41 (0.06)	0.58 (0.06)	0.52 (0.11)						
Difference	0.31 (0.08)	0.14 (0.06)	0.02 (0.06)	0.78 (0.15)						
*P* value ^b^	<.001	.02	.78	<.001						

Abbreviations: BMI, body mass index (calculated as weight in kilograms divided by height in meters squared); CF, cisgender female; CM, cisgender male; GAHT, gender-affirming hormone treatment; HDL, high-density lipoprotein; SBP, systolic blood pressure; TF, transfeminine; TM, transmasculine.

SI conversion factors: To convert HDL to millimoles per liter, multiply by 0.0259; glucose to millimoles per liter, multiply by 0.0555.

^a^ Between-group comparisons.

^b^ Within-group comparisons.

^c^ Natural log transformation of triglycerides.

**Figure 1.**
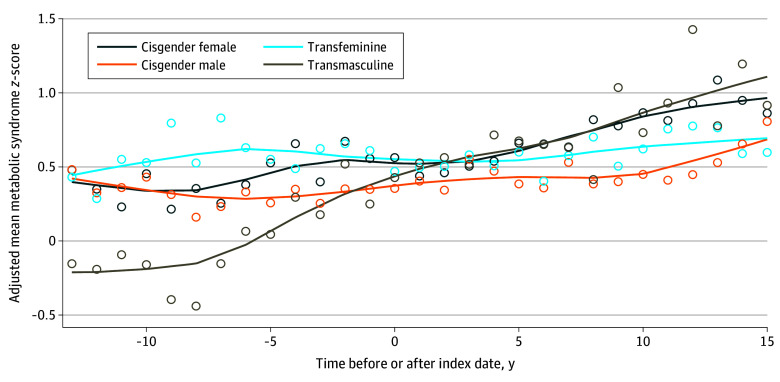
Adjusted Metabolic Syndrome z-Scores of Transgender and Cisgender Veterans After vs Before the Index Date With Smoothing Data were adjusted for time and age. Data markers represent mean z-scores and lines represent trends.

**Figure 2.**
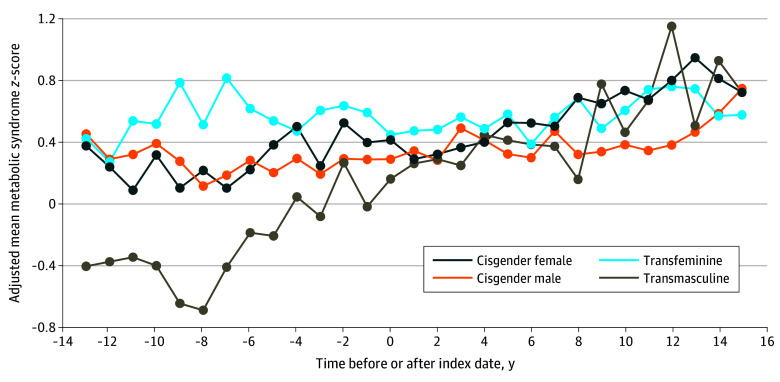
Adjusted Metabolic Syndrome z-Score of Transgender and Cisgender Veterans Before and After the Index Date Without Smoothing Data were adjusted for time and age. Data markers represent mean z-scores and lines represent trends.

The smoothed and unsmoothed longitudinal alterations in metabolic syndrome z-scores as time- and age-adjusted mean scores are presented in Figure 1 and Figure 2. Notably, the transmasculine group began with the lowest metabolic syndrome z-score of all groups, with an increase to the highest score at the final time point. The transfeminine and cisgender male groups, both of which were assigned male at birth, had higher metabolic syndrome z-scores at the initial time point; while the cisgender male z-score increase moderately with time, the transfeminine group did not show substantial changes. The individuals who received GAHT experienced the greatest changes in metabolic syndrome z-score values, with testosterone treatment leading to the greatest increase in z-score (in transmasculine individuals) and estradiol treatment leading to the smallest change in z-score (in transfeminine individuals). In a second model, all the metabolic syndrome components and metabolic syndrome z-scores were adjusted for age to account for a possible decrease in sex hormone levels in the cisgender individuals with aging. The Spearman correlation coefficient of age with metabolic syndrome z-score ranged from 0.095 to 0.195 across the transgender and cisgender groups, which indicated a very low positive correlation. Controlling for the effect of group and surveillance time and the random effect of participant, the coefficient (*r*) for the correlation of age with the (residual) metabolic syndrome z-score was 0.034. The adjustment for age did not change the overall results (Table 3).

**Table 3.** Age-Adjusted Within-Group Changes Before and After GAHT and Between-Group Differences in Within-Group Change

Metabolic syndrome component	Change, mean (SEM)				Difference-in-difference *P* value^a^					
	CF	CM	TF	TM	CF vs CM	CF vs TF	CF vs TM	CM vs TF	CM vs TM	TF vs TM
**BMI**										
Before GAHT	28.5 (0.5)	29.2 (0.4)	29.7 (0.4)	28.3 (1.0)	.31	.001	.24	.01	.08	.004
After GAHT	29.9 (0.4)	30.3 (0.4)	30.1 (0.3)	30.5 (0.8)						
Difference	1.4 (0.3)	1.1 (0.2)	0.3 (0.2)	2.3 (0.6)						
*P* value^b^	<.001	<.001	.12	<.001						
**HDL level, mg/dL**										
Before GAHT	45.6 (1.1)	44.7 (0.8)	42.7 (0.9)	53.4 (2.2)	.17	<.001	<.001	.002	<.001	<.001
After GAHT	48.5 (0.9)	49.2 (0.8)	50.0 (0.7)	49.6 (1.8)						
Difference	2.9 (0.9)	4.4 (0.7)	7.3 (0.7)	−3.8 (1.8)						
*P* value^b^	.001	<.001	<.001	.03						
**Log triglycerides, mg/dL** ^c^										
Before GAHT	4.85 (0.05)	4.88 (0.03)	4.97 (0.04)	4.56 (0.09)	.12	.003	.01	.11	<.001	<.001
After GAHT	4.92 (0.04)	4.86 (0.03)	4.89 (0.03)	4.84 (0.07)						
Difference	0.07 (0.04)	−0.02 (0.03)	−0.09 (0.03)	0.29 (0.08)						
*P* value^b^	.11	.62	.006	<.001						
**Blood glucose level, mg/dL**										
Before GAHT	100.1 (3.4)	98.9 (2.4)	104.1 (2.7)	99.5 (6.5)	.03	.63	.048	.06	.46	.08
After GAHT	111.0 (2.4)	119.2 (2.2)	117.1 (2.0)	124.8 (4.9)						
Difference	10.9 (3.5)	20.2 (2.6)	13.0 (2.7)	25.3 (6.4)						
*P* value^b^	.001	<.001	<.001	<.001						
**SBP, mm HG**										
Before GAHT	125.8 (1.1)	124.6 (0.7)	127.2 (0.9)	120.6 (2.1)	.01	.10	.04	<.001	.59	.001
After GAHT	127.3 (0.7)	129.6 (0.7)	126.4 (0.6)	126.8 (1.5)						
Difference	1.5 (1.1)	5.1 (0.8)	−0.8 (0.9)	6.3 (2.1)						
*P* value^b^	.17	<.001	.37	.002						

Abbreviations: BMI, body mass index (calculated as weight in kilograms divided by height in meters squared); CF, cisgender female; CM, cisgender male; GAHT, gender-affirming hormone treatment; HDL, high-density lipoprotein; SBP, systolic blood pressure; SEM, standard error of the mean; TF, transfeminine; TM, transmasculine.

SI conversion factors: To convert HDL to millimoles per liter, multiply by 0.0259; glucose to millimoles per liter, multiply by 0.0555.

^a^ Between-group comparisons.

^b^ Within-group comparisons.

^c^ Natural log transformation of triglycerides.

### Estimation of Future Risk

The metabolic syndrome z-score can be used to estimate the risk of future CVD and T2D. In cohorts of a study by Gurka et al,^9^ each 1-SD increase in metabolic syndrome z-score (BMI-based) was associated with a significant increase in the risk of CVD (hazard ratio [HR], 1.78; 95% CI, 1.67-1.90) and T2D (HR, 3.37; 95% CI, 3.15-3.61). Based on this association and metabolic syndrome z-score values expressed in SDs, the transmasculine group in our study, which had a mean (SEM) difference after vs before transition of 0.78 (0.15; *P* < .001) (Table 2), would have an HR for CVD of 1.58 and for T2D of 3.67 (95% CIs are not presented because they would not take into account the covariance and therefore might not be representative of the true 95% CIs). For the transfeminine group, the calculated HR for CVD would be 1.10, while that for T2D would be 1.22. For the cisgender female group, the HR for CVD would be 1.25 and the HR for T2D would be 1.59. For the cisgender male group, the HR for CVD would be 1.21 and the HR for T2D would be 1.48.

## Discussion

Metabolic syndrome is associated with an increased risk of ASCVD, insulin resistance, T2D, systolic hypertension, and NAFLD.^5,6,28,29,30,31^ Previous studies regarding the effect of GAHT on metabolic syndrome have been limited to analyses of individual metabolic syndrome components.^32,33,34,35,36^ In the current study, we investigated the association of GAHT with each component of metabolic syndrome and additionally assessed the association of GAHT with the overall metabolic syndrome z-score using the methods reported previously by Gurka et al.^9^ The metabolic syndrome z-score that was used in this study also provides an estimate of the risk for diabetes and cardiovascular outcomes.^37^

Traditional metabolic syndrome assessment strategies are binary^38^ and are not well suited to evaluating a patient’s risk over time. Gurka and colleagues’ BMI-based z-score^9^ was developed using the 1999-2010 National Health and Nutrition Survey. It was then validated and compared with the continuous-risk z-score for metabolic syndrome using waist circumference among 13 094 participants in the Atherosclerosis Risk in Communities Study and Jackson Heart Study and was found to be comparable to the score using waist circumference as a means of detecting the severity of metabolic syndrome and estimating the risk of diabetes and CVD outcomes.^7,8,9,37,38^ Due to a lack of waist circumference data in our study, we applied Gurka and colleagues’ metabolic syndrome BMI-based z-score^9^ using BMI, SBP, HDL, blood glucose, and log triglycerides.

We conducted longitudinal analyses incorporating data from more than 28 years. In comparisons of pre- and posttransition mean z-scores for study participants, we observed a worsening of metabolic syndrome components in the transmasculine group after transition, with increases in BMI, SBP, blood glucose level, and log triglyceride level together with reductions in HDL level. In contrast, the transfeminine group experienced improvements in several components, including decreased SBP and triglyceride levels together with increased HDL levels and no significant change in BMI or blood glucose level. It is necessary to note that transgender individuals receiving GAHT could experience changes in their muscle mass that could contribute to changes in BMI. However, they also experience a change in the pattern of fat distribution to match their affirmed gender.^39^ The change in fat distribution pattern in transmasculine individuals to a more central pattern could contribute to an increase in triglycerides and fatty acids.^40^

Our results showed an upward trend in metabolic syndrome z-scores for transmasculine individuals; however, for the other 3 groups, the z-score lines were relatively flat, thereby reflecting less change in the metabolic syndrome z-score in these groups. That said, the trend lines for the adjusted mean metabolic syndrome z-scores for these 3 groups suggest higher metabolic syndrome z-scores than in the transmasculine group until follow-up year 10; tailored care plans for transfeminine compared with transmasculine veterans should incorporate the possibility of differential adjustment over time following GAHT initiation. Our hypothesis that sex hormones are associated with metabolic syndrome appeared to be valid in this cohort with the exception of cisgender females. Cisgender female veterans had metabolic syndrome z-scores higher than 0 at baseline, and their longitudinal z-score change over time surpassed that for transfeminine and cisgender male veterans. This is not fully consistent with our hypothesis that ovarian sex hormones would be protective. It has been documented that cisgender female veterans have more complex comorbidities than cisgender female civilians, including a greater prevalence of posttraumatic stress disorder and other mental health disorders, military sexual trauma, lifetime and military interpersonal trauma, and history of childhood sexual abuse, which may place them at a higher risk of worse clinical outcomes.^41,42^

Our results regarding the associations of GAHT with metabolic syndrome components are in agreement with and extend on findings of previous studies in several respects: (1) through the inclusion of a metabolic syndrome z-score, (2) through comparisons with cisgender groups, and (3) through the longer follow-up time frame than in previous studies. For example, in a 2019 prospective study by Van Velzen et al^32^ that assessed 188 transmasculine and 242 transfeminine individuals before and 1 year after GAHT initiation, transmasculine participants experienced unfavorable changes in lipid profiles, whereas transfeminine participants experienced favorable changes, and no changes in BP occurred in either group. In 2017, Maraka et al^33^ performed a meta-analysis of 29 eligible studies that included 3231 transfeminine and 1500 transmasculine individuals. These authors reported that GAHT in transmasculine persons was associated with increased serum triglyceride and low-density lipoprotein levels after 24 months of treatment, whereas HDL levels decreased. Smaller studies (<50 persons in each group) also reported that transfeminine individuals experienced more favorable changes in metabolic syndrome components following GAHT than did transmasculine individuals.^34,35,36^

We also observed that individuals with exogenous testosterone administration (transmasculine) had higher metabolic syndrome z-scores than those with endogenous male hormones (cisgender male). Similarly, individuals with exogenous estradiol administration (transfeminine) had lower metabolic z-scores than those with endogenous female hormones (cisgender female) (Table 2). This raises the possibility that the action of exogenous hormones is influenced by chromosomal sex and/or organizational effects of gonadal hormones that occurred during development.^24^ We also derived methodologic information that is relevant for studies of GAHT effects, including the importance of validating the classification of gender dysphoria and determining the date of GAHT initiation and the value of harvesting longitudinal data. We determined that medical record review may be a critical step in assessing the association of GAHT with clinical outcomes. In particular, we found that *ICD-9* and *ICD-10* codes are not reliable as the only identifiers of gender dysphoria and that the date of GAHT initiation is key to assessing clinical outcomes before and after hormonal transition.

### Limitations

As with most studies of this type, there are limitations. We were unable to draw causal inferences from this observational study. The limited sample size of the transmasculine and cisgender female groups may have led to an attenuated effect size, and we acknowledge that veterans, especially female veterans, are not representative of the general population due to historical patterns of military recruitment and deployment and sociological factors driving service in the military; in addition, veterans in the VHA differ from those not using the VHA.^43^ We did not take into account minority stress among transgender veterans, which is associated with health and well-being outcomes.^44^ Future studies should validate the correlation of the metabolic syndrome z-score with incident ASCVD, NAFLD, and T2D; document metabolic syndrome z-scores in transgender persons using waist circumference to account for lean vs fat mass; and explore variation in metabolic syndrome z-scores based on gender identity rather than sex assigned at birth, as changing sex hormones at some point in adult life is likely to have unique effects on health status.

## Conclusions

Our data from this cohort study indicated that in both cisgender and transgender individuals, estradiol was associated with reduced metabolic syndrome risk, whereas testosterone was associated with increased risk. These findings are relevant for the management of metabolic syndrome risk factors in cisgender and transgender individuals. It is also important to note that exogenous sex hormones are not equivalent to endogenous sex hormones, nor is the body in which they operate the same as one never exposed to the opposing sex hormones.

## Notes

Supplement 1.
eMethods.
**eFigure.** Cohort Construction **eTable.** Surveillance Time Statistics

Supplement 2.
Data Sharing Statement
