# Supplementary material for: Gender-Affirming Hormone Treatment and Metabolic Syndrome Among Transgender Veterans
Source: JAMA Netw Open. 2024 Jul 2;7(7):e2419696. doi: 10.1001/jamanetworkopen.2024.19696 (PMC11220566; doi:10.1001/jamanetworkopen.2024.19696)
Supplement: Supplement 1. — eMethods. eFigure. Cohort Construction eTable. Surveillance Time Statistics [file jamanetwopen-e2419696-s001.pdf]

Supplementary Online Content

Hashemi L, Marijic-Buljubasic A, Budoff MJ, et al. Gender-affirming hormone treatment and metabolic syndrome among transgender veterans. *JAMA Netw Open*. 2024;7(7):e2419696. doi:10.1001/jamanetworkopen.2024.19696

eMethods
eFigure. Cohort Construction
eTable. Surveillance Time Statistics

This supplementary material has been provided by the authors to give readers additional information about their work.

© 2024 Hashemi L et al. *JAMA Network Open*.

eMethods

Of 11,011 individuals with relevant ICD codes diagnoses of gender identity disorder, transsexualism, gender dysphoria, and/or transgender status, 4,586 had an estrogen prescription filled at VA while 1,451 had a testosterone prescription. Of the remaining subjects, 4,595 did not have a hormone (either estrogen or testosterone) prescription filled at any VA, while 379 had prescriptions for both estrogen and testosterone filled at some point. We evaluated the sex provided by CDW for each patient against the prescription hormones that they received to identify TM and TF status. The Provided sex for 4,586 individuals on estrogen was male such that members of this group were identified as TF for the purposes of this study, while the sex for 1,451 individuals on testosterone was female such that members of this group were referred to as TM. We were not able to specify the gender status for the remainder of the cohort (379 individuals who had a prescription for both estrogen and testosterone as well as 4,595 individuals with no hormone prescription record at VHA) It is important to note that the sex provided in the CDW, which is noted in this dataset as “gender” even though it refers to sex, is self-identified by veterans and, in some cases, may not correspond to their biological assigned sex at birth. The VHA began providing medical therapy to transgender veterans in 2006. Prior to that, providing GAHT was at the discretion of local endocrine physicians and pharmacies at each site.

In order to clarify this data, we obtained permission from VA GLA IRB to access the patients' medical records.
We used Compensation and Pension Record Interchange (CAPRI) and Voogle to review patients' medical record. CAPRI is an automated VHA information system that provides authorized users read-only access to Veterans' medical records and it is also linked to Joint Legacy Viewer (JLV) . JLV contains medical data from Department of Defense and also community care.
Voogle is a VHA web-based application that enables searching clinical notes and many structured data domains for all care a patient has received in the VHA.
We reviewed 999 charts (all patients with last names beginning with letter A or B) out of 11,011 veterans with relevant ICD codes for gender identity disorder. Using Voogle and CAPRI, extracting information including gender status (TM vs. TF), date of hormone initiation (index date), For individuals receiving hormone prescriptions outside of the VHA (e.g., through community medical practices or from the Department of Defense), we accessed JLV to obtain the hormone formulation, dose, and patient hormone levels. of 999 veterans only 789 patients (78.9%) had gender dysphoria, The assignment of ICD codes for gender dysphoria in the other 210 individuals may have occurred because they revealed that a family member is a transgender individual or discussed he topic in a therapy session. Of the 789 individuals with confirmed gender dysphoria, 645 (81.7%) had undergone hormonal transition and were currently on GAHT (151 TM and 494 TF veterans).
We confirmed the date of GAHT initiation (index date) for 636 individuals by reviewing primary care, endocrine, and mental health social worker notes for these subjects. The first hormone prescriptions filled at VHA facilities for 226 individuals were the same as the index date, while the remainder of these 636 individuals began GAHT prior to having their first prescription filled at the VHA. If the GAHT initiation date was not available, we used the first hormone prescription at any VHA facility as the index date (9 individuals).

© 2024 Hashemi L et al. *JAMA Network Open*.

Supplemental Figure 1: Cohort Construction

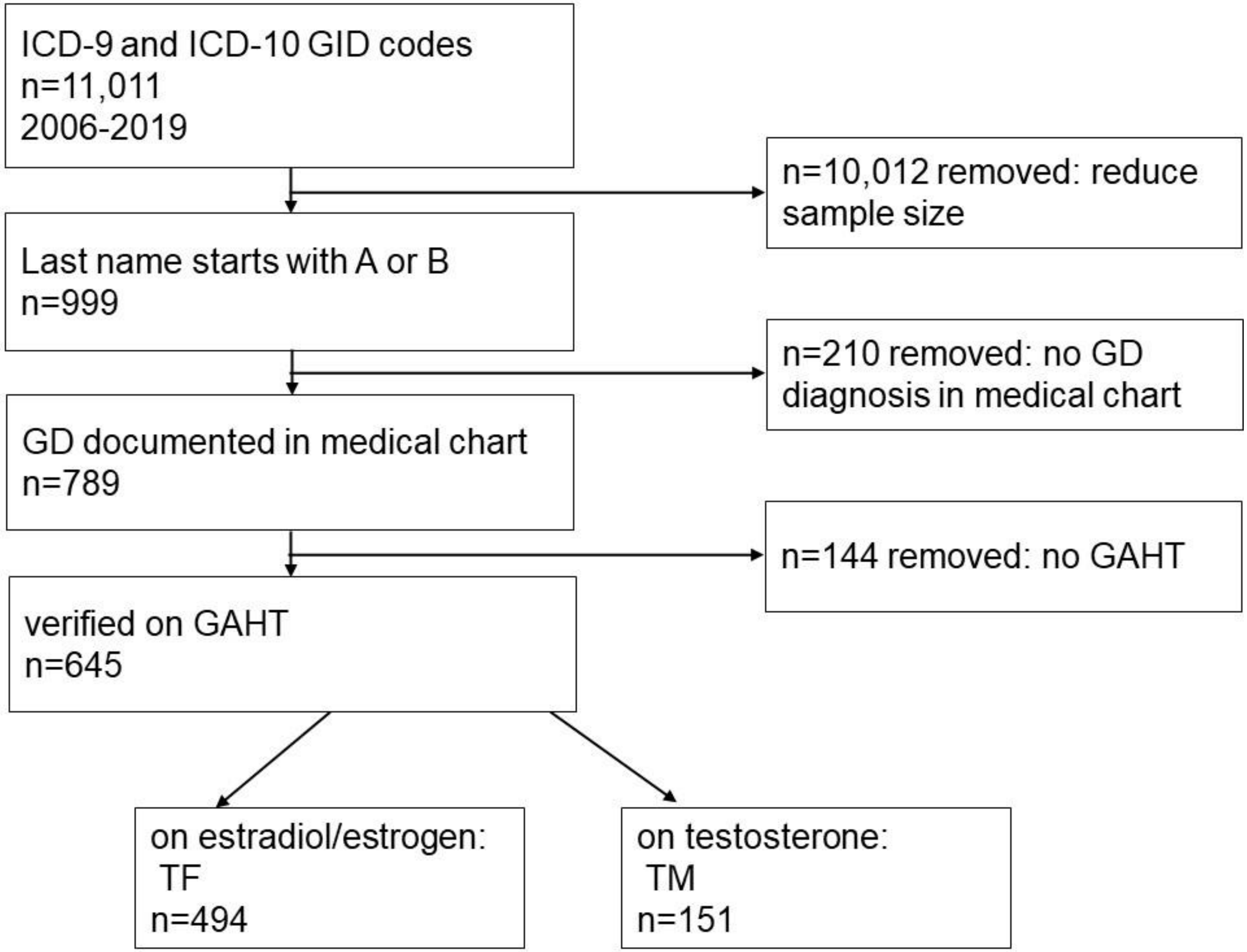

ICD: International Classification of Diseases, GID: Gender identify disorders, GD: Gender dysphoria, GAHT: Gender-affirming hormone treatment. TF: transfemale, TM: transmale.

© 2024 Hashemi L et al. *JAMA Network Open*.

eTable. Surveillance time statistics April, 2024

up to 13 years before transition, up to 15 years after transition - span of 28 years

number observations per person

Group	total n	n > one observation	min	Q1	median	mean	Q3	max	SD
C-F	279	273	1.0	2.0	5.0	6.8	10.0	28.0	5.6
C-M	365	360	1.0	3.0	5.0	7.1	10.0	39.0	6.3
T-F	495	327	1.0	3.0	7.0	8.6	12.0	30.0	6.4
T-M	151	110	1.0	3.0	5.0	6.3	9.0	34.0	5.4
overall	1290	1070	1.0	3.0	6.0	7.4	11.0	39.0	6.1

years of surveillance from pre-transition to post-transition

Group	total n	n > one observation	min	Q1	median	mean	Q3	max	SD
C-F	279	273	1.0	2.0	6.0	6.5	11.0	15.0	4.6
C-M	365	360	1.0	2.0	6.0	6.7	11.0	15.0	4.5
T-F	495	327	1.0	3.0	7.0	7.4	11.0	15.0	4.4
T-M	151	110	1.0	2.0	6.0	6.2	9.0	15.0	4.4
overall	1290	1070	1.0	3.0	6.0	6.8	11.0	15.0	4.5

CF: cisfemale, CM: cismale, TF: transfemale, TM: transmale, n: number, Q1=first quartile, Q3=third quartile

© 2024 Hashemi L et al. *JAMA Network Open*.
