# Supplementary material for: Gender-Affirming Hormone Treatment and Metabolic Syndrome Among Transgender Veterans
Source: JAMA Netw Open. 2024 Jul 2;7(7):e2419696. doi: 10.1001/jamanetworkopen.2024.19696 (PMC11220566; doi:10.1001/jamanetworkopen.2024.19696)
Supplement: Supplement 2. — Data Sharing Statement [file jamanetwopen-e2419696-s002.pdf]

Data Sharing Statement

Hashemi. Gender-Affirming Hormone Treatment and Metabolic Syndrome Among Transgender Veterans. *JAMA Netw Open*. Published July 02, 2024.
doi:10.1001/jamanetworkopen.2024.19696

Data
Data available: Yes
Data types: Deidentified participant data
How to access data: The datasheet will be shared upon request after obtaining permission from VHA,
When available: With publication

Supporting Documents
Document types: Statistical/analytic code
How to access documents: The request can be sent to lhshemi@mednet.ucla.edu
When available: With publication

Additional Information
Who can access the data: researchers whose proposed use of the data has been approved
Types of analyses: for a specified purpose
Mechanisms of data availability: after approval of a proposal, or with a signed data access agreement).
Any additional restrictions: Obtaining data from VHA for non-VA employee is very hard and needs approval.
